# SURVIVING THE COMPLEXITY: USING GROUNDED THEORY TO UNDERSTAND KINSHIP CAREGIVING

**DOI:** 10.1093/geroni/igz038.2956

**Published:** 2019-11-08

**Authors:** Catherine J Tompkins

**Affiliations:** George Mason University, Fairfax, Virginia, United States

## Abstract

The role of a caregiver often goes beyond the task of caring for someone who is dependent in managing activities of daily living. Children are dependent on others to care for them due solely to their age and maturity; others are dependent due to chronic ailments or short-term disabilities. Regardless of why someone is dependent, the caregiving relationship is complex. This paper focuses on a grounded theory, developed and applied to understand the complexities of kinship caregiving. The literature continues to support the identified needs of kinship caregivers (Tompkins, 2015; Lee, Clarkson-Hendrix, & Lee, 2016). To understand the unique needs of kinship families, the following grand tour question was asked: What is it like for you to live within a kinship caregiving household? The theory was developed over several years based on observational data and 15 interviews with grandparent caregivers and at least one of the grandchildren they were raising. The theory, Surviving the Complexity, is a survival process of taking on the caregiving role and doing one’s best in spite of multiple obstacles. Surviving the complexity consists of three stages: rescuing, taking-on and role reversal. The theory identifies and explains emotional, relationship and situational complexity within kinship families. Hope and denial are factors of emotional complexity: “It’s not that she (my daughter) does not love him (the child), she is just unable to right now. She will get better.” Theory development and further application of the theory will be discussed.

